# Comparative transcriptomic and proteomic analyses of two salt-tolerant alfalfa (*Medicago sativa* L.) genotypes: investigation of the mechanisms underlying tolerance to salt

**DOI:** 10.3389/fpls.2024.1442963

**Published:** 2024-11-13

**Authors:** Jiahui Hang, Ting Song, Ling Zhang, Wenjun Hou, Xiaoxia Liu, Dongmei Ma

**Affiliations:** ^1^ Breeding Base for State Key Laboratory of Land Degradation and Ecological Restoration in Northwest China, Ningxia University, Yinchuan, China; ^2^ Ministry of Education Key Laboratory for Restoration and Reconstruction of Degraded Ecosystems in Northwest China, Ningxia University, Yinchuan, China; ^3^ Key Laboratory of Modern Molecular Breeding for Dominant and Special Crops in Ningxia, Ningxia University, Yinchuan, China

**Keywords:** alfalfa, transcriptome, proteome, salt tolerance, molecular mechanism

## Abstract

Abiotic stressors such as salt stress restrict plant development and output, which lowers agricultural profitability. In this study, alfalfa (*Medicago sativa* L.) varieties with different levels of salt tolerance were examined using high-throughput RNA sequencing (RNA-Seq) and Tandem Mass Tags (TMT) technologies to study the reactions of the root systems to salt stress, from transcriptomics and proteomics perspectives. The varieties Atlantic (AT) and Zhongmu-1 (ZM-1) were selected and evaluated after 2 h and 6 h of treatment with 150 mM NaCl. The results showed that under salt stress for 2 h, 1810 differentially expressed genes (DEGs) and 160 differentially expressed proteins (DEPs) in AT were screened, while 9341 DEGs and 193 DEPs were screened in ZM-1. Under salt stress for 6 h, 7536 DEGs and 118 DEPs were screened in AT, while 11,754 DEGs and 190 DEPs were screened in ZM-1. Functional annotation and pathway enrichment analyses indicated that the DEGS and DEPs were mainly involved in the glutathione metabolism, biosynthesis of secondary metabolites, glycolysis/gluconeogenesis, carbon fixation in photosynthetic organisms, and photosynthesis pathways. A series of genes related to salt tolerance were also identified, including *GSTL3* and *GSTU3* of the *GST* gene family, *PER5* and *PER10*, of the *PER* gene family, and proteins such as *APR* and *COMT*, which are involved in biosynthesis of secondary metabolites. This study provides insights into salt resistance mechanisms in plants, and the related genes and metabolic pathways identified may be helpful for alfalfa breeding in the future.

## 1 Introduction

Alfalfa (*Medicago sativa* L.) is a perennial herbaceous legume and one of the world’s most significant feed crops. Alfalfa exhibits remarkable tolerance to cold temperatures, salinity, and drought. Its primary root system possesses strong nitrogen-fixing capabilities, which contribute to soil loosening, promote the accumulation of organic matter and total nitrogen, improve soil structure, and enhance soil fertility. Compared to other crops, alfalfa offers a high yield and protein content, exhibits good palatability, and is rich in various amino acids essential for the growth and development of animals. Additionally, it contains polysaccharides and flavonoids, as well as calcium, phosphorus, and other trace elements, making it a high-quality pasture grass (Chu and Zhang, 2022; Du et al., 2021). Although alfalfa exhibits some resistance to salt stress, the intensification of soil salinization, coupled with the rising demand for pasture in animal husbandry, has rendered it crucial to maintain the quality and yield of alfalfa under such conditions. Thus, ensuring high-efficiency alfalfa production in order to achieve various ecological benefits has emerged as an urgent issue that requires immediate attention.

Salt stress is a significant abiotic stress that adversely impacts crop yield. A study by the Food and Agriculture Organization of the United Nations (FAO) indicated that over 1 billion hectares of farmland are affected by salinity. Approximately 25-33% of the world’s irrigated land suffers from secondary salinity. As the concentration of salt ions in the soil increases, water uptake by the plant root system is impeded and this triggers a series of physiological and biochemical responses within the plant. These responses include effects on seed germination, photosynthesis, ionic balance, and cell structure. Salt stress promotes the synthesis of abscisic acid, induces stomatal closure, and reduces CO_2_ uptake by plants (Huang et al., 2021). Additionally, it damages photosynthesis by decreasing the content of photosynthetic pigments and hinders the photosynthetic electron transfer process. Salt stress further disrupts the ionic balance within the plant, leading to ionic toxicity (Shahid et al., 2020). Excess Cl^-^ disrupts the membrane system and negatively affects the chlorophyll content, resulting in leaf senescence and yellowing. Moreover, significant accumulation of Na^+^ inhibits the uptake of K^+^ and Ca^2+^ by the plant. Malondialdehyde (MDA) serves as a critical indicator for evaluating damage to membrane structures. Salt stress triggers the generation of substantial quantities of reactive oxygen species (ROS), resulting in membrane lipid peroxidation, which compromises the integrity of the cell membrane (Tsikas, 2017). Under salt stress, a large amount of ROS (O^2-^, H_2_O_2_, OH^-^) accumulate in cells, damaging the cell structure and affecting intracellular biochemical reactions (Ren et al., 2021). Plants use a variety of mechanisms to respond to salt stress and, therefore, there are many possible pathways through which stress tolerance can be manipulated and improved. For example, plants have evolved powerful antioxidant systems, including superoxide dismutase (SOD), catalase (CAT), peroxidase (POD), and glutathione (GSH) (Kapoor et al., 2019). Identifying and understanding the genes and molecular mechanisms that mediate a plant’s response to salinity stress is crucial for enhancing the plant’s salt tolerance. This would ultimately contribute to the development of saline-resistant crops and improved food production.

Based on RNA sequencing (RNA-Seq) data, numerous significant biotic and abiotic stress-responsive genes have been characterized. A number of genes have been mined through RNA-Seq as candidate salt tolerance genes for genetic editing, including ERF (Xiao et al., 2024), WRKY (Qin et al., 2015), bHLH (Ahmad et al., 2015), and bZIP (Xu et al., 2023). Many of these transcription factors play a crucial role in regulating the expression of target genes and metabolic pathways, thereby significantly contributing to our understanding of the mechanisms underlying salt tolerance in plants.

The Tandem Mass Tags (TMT) technique, which has the advantages of high throughput, good reproducibility, accurate quantification, and high resolution, has been successful in the identification and quantification of proteins in monocotyledons to higher woody plants. Cai et al. analyzed the salt tolerance of *Lonicera japonica* by the TMT technique and found that the expression levels of genes related to the phenylpropanoid biosynthesis and monoterpene biosynthesis pathways were up-regulated, and the differential proteins were mainly involved in the energy metabolism and carbohydrate metabolism pathways, promoting enhanced salt tolerance (Cai, 2022). Zeng et al. revealed the key factors in alfalfa’s response to waterlogging stress (Zeng et al., 2019). With the continued development of biotechnology, protein quantitative analysis technology, as a high-throughput screening technique commonly used in quantitative proteomics, has now received attention in various fields and is of great significance for molecular biology research and development.

According to previous experimental studies, when alfalfa was subjected to salt stress for a short period of time, the wilting phenomenon was more obvious, and when the time is too long, a series of response mentioning resistance to adversity would occur in the plant, and the phenotype would gradually stabilize. Its POD, SOD, CAT, MDA, proline (PRO), and relative water content (RWC) were all found to change. It indicated that alfalfa has a strong biochemical response to salt ion damage such as Na^+^, Cl^-^, and K^+^ within a short period of time under salt stress, therefore, short salt treatments such as: 2 h and 6 h can be studied as important nodes of salt stress in alfalfa.

Although many studies have reported the molecular mechanisms of salt stress tolerance in alfalfa, in-depth exploration of a single histology has been limited. Thus, in this study, two different salt-tolerant alfalfas (the salt-tolerant variety ZM-1 and the salt-sensitive variety AT) were compared, and a combined transcriptomics and proteomics analysis was performed. Genes that were up-regulated in the salt-tolerant variety or down-regulated in the salt-sensitive variety were hypothesized to be salt-tolerant candidate genes. This enabled the identification of key genes that significantly respond to salt stress in alfalfa. This work contributes to our understanding of the molecular mechanisms of salt tolerance in alfalfa and may contribute to alfalfa salt tolerance breeding.

## 2 Materials and methods

### 2.1 Plants, materials, and treatment conditions

Two varieties of alfalfa were selected as plant materials, the salt-tolerant cultivar ZhongMu-1 (ZM-1) and the salt-sensitive cultivar Atlantic (AT). Both varieties were provided by the Laboratory of Molecular Biology of Forage Resistance, College of Ecology and Environment, Ningxia University, Ningxia, China. ZM-1 is a salt-tolerant alfalfa variety bred in Dr. Qingchuan Yang’s laboratory, which has a high survival rate after receiving NaCl solution irrigation and is widely planted (Jin et al., 2010). AT was significantly inhibited in plant growth after salt stress and was more sensitive to salt stress compared to ZM-1.

Alfalfa seeds were first sterilized by immersion in 75% ethanol for 30 s and in 0.1% HgCl_2_ solution for 8 min. The seeds were then rinsed 3-4 times with sterile water and inoculated on sterile Petri dishes (dish size: 90 mm). Subsequently, the Petri dishes were placed in an artificial climatic chamber for germination. The temperature of the chamber was 23°C/20°C (day/night) and the chamber was kept under a 16 h/8 h (day/night) cycle with 75% humidity. Six alfalfa seedlings with similar growth conditions were selected and transferred to Hoagland nutrient solution for hydroponics (hydroponic box size: 20×20×20 cm). The nutrient solution was changed every two days to maintain freshness. After two weeks of seedling growth, the seedlings were transferred to 150 mM NaCl solution for salt treatment. The roots of the alfalfa were harvested after 0 h, 2 h, and 6 h of treatment. Treatment with distilled water served as the control. Three biological replicates were performed for each sample. The samples were flash-frozen with liquid nitrogen and preserved at -80°C.

### 2.2 Total RNA isolation and sequencing

Total RNA was isolated from the alfalfa root samples using FreeZol Reagent (Vazyme, Nanjing, China); all operations were carried out on ice. RNA quality was checked on 1% agarose gel without RNase and the RNA concentration was determined using a microvolume spectrophotometer (Colibri LB 915; Berthold, Germany). An Agilent 2100 Bioanalyzer (Agilent Technologies, Santa Clara, CA, USA) was used to assess the RNA integrity and the presence of DNA contamination in the samples. The cDNA library was constructed using a HiScript III First Strand cDNA Synthesis Kit (Vazyme, Nanjing, China). Samples were stored at -20°C for sequencing.

### 2.3 Transcriptomic and proteomic analysis

Shanghai Ouyi Biotechnology Co., Ltd. (Shanghai, China) performed the RNA-seq for this experiment. A total of 18 cDNA libraries were constructed. High-throughput sequencing was performed on the pre-obtained libraries with the help of an Illumina Novaseq 6000. Sequences of a double-end size of 150 bp were produced, and the reads with substandard quality were eliminated by FASTP software. High- quality clean reads were selected for further analyses. Gene expression was calculated using the Fragments Per Kilobase per Million mapped reads (FPKM) method.

Proteomic analysis was performed by Shanghai Lumine Biotechnology Co., Ltd (Shanghai, China) using TMT, an *in vitro* labelling technique developed by Thermo. This technique is widely used in studies analyzing differentially expressed proteins (DEPs). The basic procedure for the TMT quantitative proteomics experiment was as follows. First, the total proteins were extracted from the samples. Then, the total protein concentration was determined by a bicinchoninic acid (BCA) assay. Next, the mass was tested by sodium dodecyl sulfate polyacrylamide gel electrophoresis (SDS-PAGE). Another portion was taken for trypsin digestion and labelling. Then, an equal amount of each labelled sample was taken and mixed for chromatographic separation. Finally, the samples were subjected to Liquid Chromatography-Mass Spectrometry (LC-MS/MS) and statistical analysis.

### 2.4 Statistical analysis

Differentially expressed genes (DEGs) were screened by comparing the data between different samples. Analysis was performed using DESeq. In the process of DEG detection, a Fold Change (FC) ≥ 2 and *p*-value < 0.05 were used as the screening criteria. Gene Ontology (GO) and Kyoto Encyclopedia of genes and genomes (KEGG) analyses of the DEGs were performed based on hypergeometric algorithmic distributions.

Proteomic analyses were performed using TMT technology. Credible proteins were screened according to the following criteria: Score Sequest HT > 0 and unique peptide ≥ 1; blank values removed. The FC and *p*-values of the comparison groups were calculated. Significant proteins were screened according to the following criteria: FC > 1.5 and *p*-value < 0.05. GO and KEGG analyses of the DEPs were performed based on hypergeometric algorithmic distributions.

### 2.5 Quantitative real-time PCR validation

To validate the RNA-seq data, four genes were randomly selected for quantitative RT-PCR analysis. Primers used for qRT-PCR validation are listed in **Supplementary Table S1**. The *β-ACTIN* gene of *Medicago sativa* was used as a control, and each group was replicated three times. The relative expression of the candidate genes was calculated using the 2^-ΔΔCt^ method.

## 3 Results

### 3.1 Analysis of DEGs

**Figure 1:**
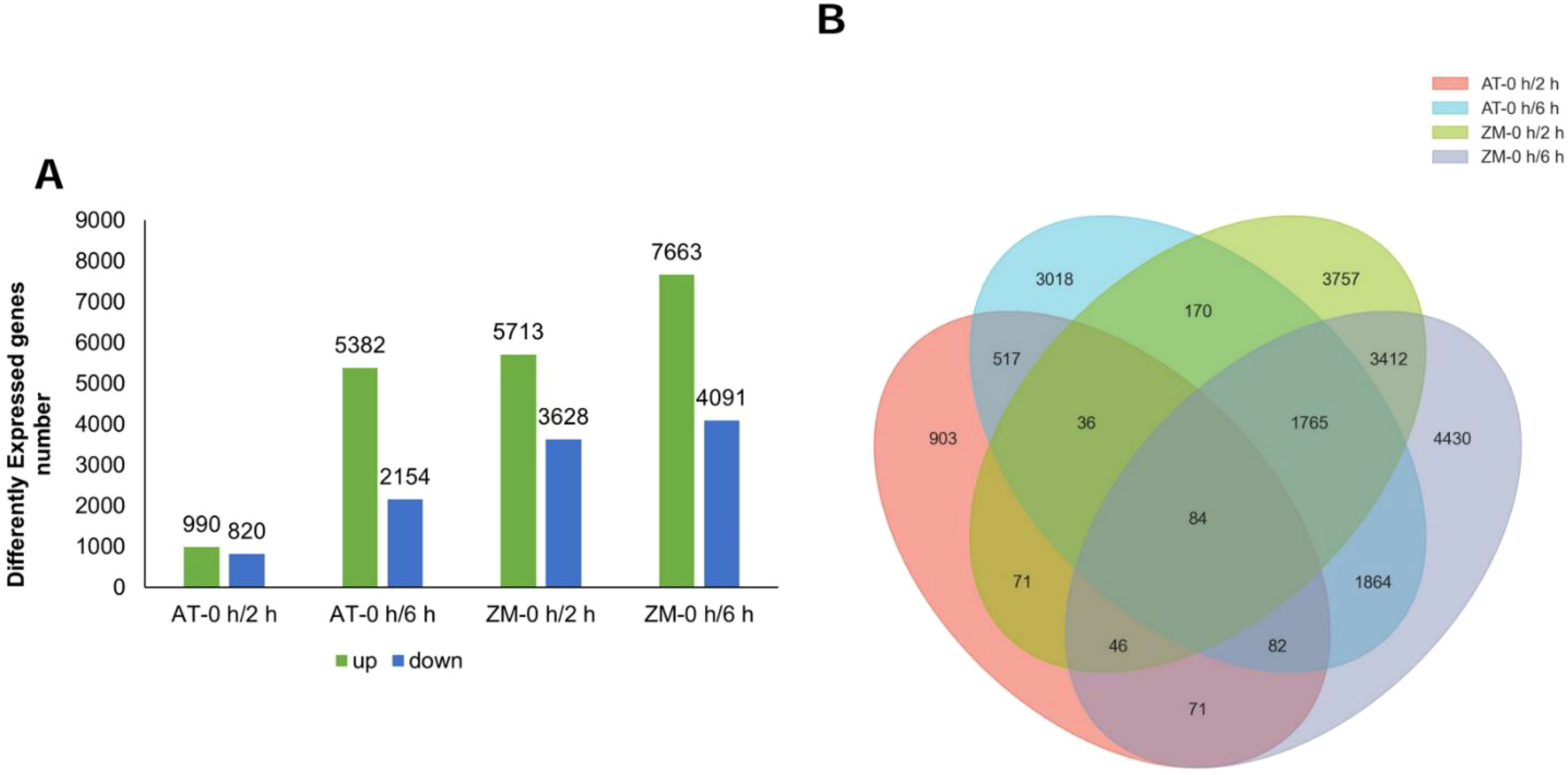
Analysis of DEGs. **(A)**The number of up-regulated and down-regulated genes in the four groups. **(B)** The numbers of overlapping genes among the four groups. AT-0 h/2 h and AT-0 h/6 h represent the comparison groups of the genes identified at 2 h and 6 h after treatment to 0 h in Atlantic. ZM-0 h/2 h and ZM-0 h/6 h represent the comparison groups of the genes identified at 2 h and 6 h after treatment to 0 h in Zhongmu-1.

RNA-seq was used to examine the DEGs between the salt-tolerant and salt-sensitive varieties after 2 h and 6 h of salt stress. The numbers, changes, and overlaps in the DEGs identified in the four groups (AT-0 h/2 h, AT-0 h/6 h, ZM-0 h/2 h, ZM-0 h/6 h) are shown in a Venn gram (**Figure 1**). After 2 h of salt stress, 1810 DEGs were screened in AT and 9341 DEGs were screened in ZM-1. The number of up-regulated DEGs was higher than the number of down-regulated DEGs in both varieties. After 6 h of salt stress, 7536 DEGs were detected in AT, among which, 5382 of the DEGs were up-regulated and 2154 were down-regulated. Further, 11,754 DEGs were detected in ZM-1, among which, 7663 were up-regulated and 4091 were down-regulated. Only 84 DEGs were common to both varieties under salt stress.

### 3.2 Analysis of common DEGs

**Figure 2:**
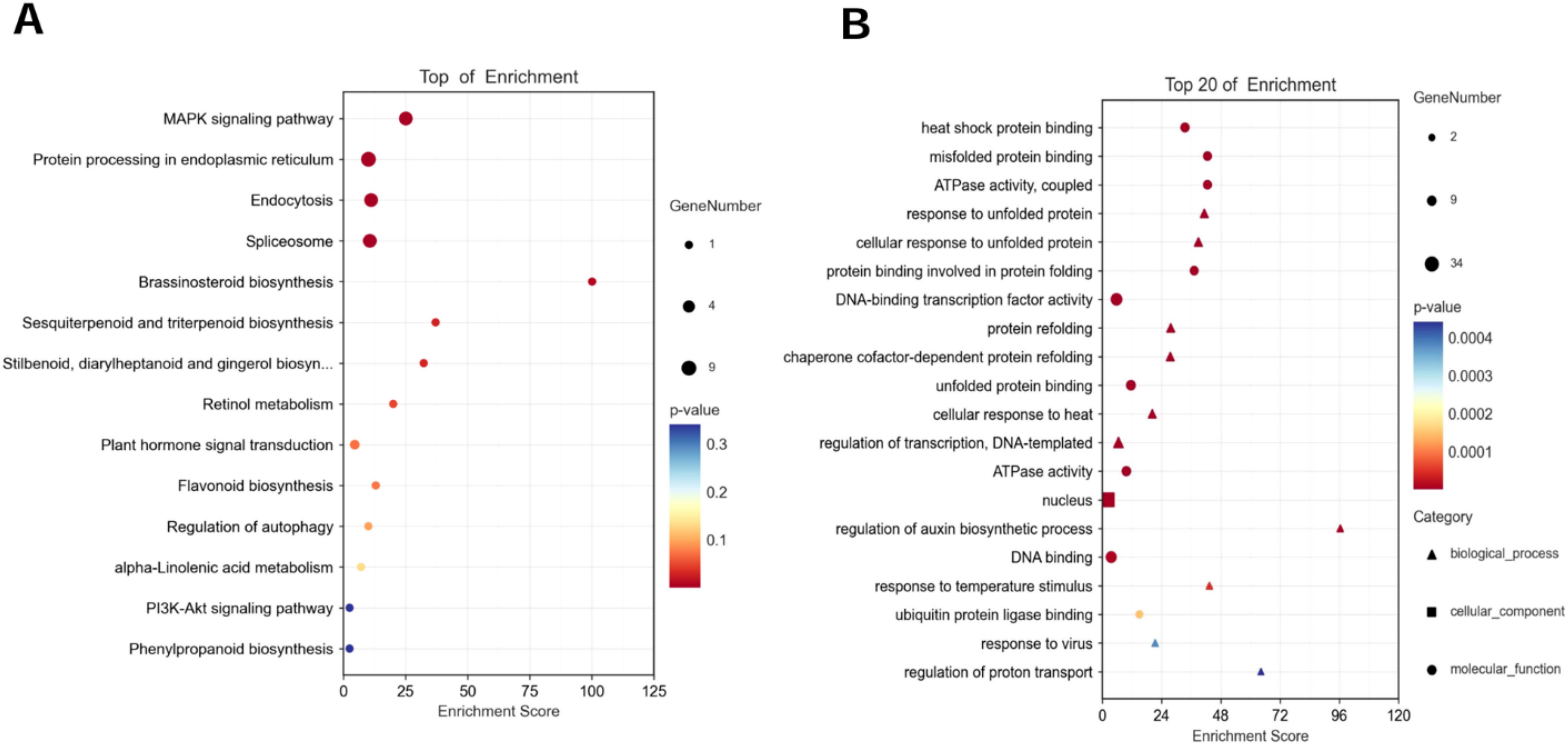
Functional analysis of DEGs. **(A)** KEGG analysis of common DEGs in AT-0 h/2 h, 6 h and ZM-0 h/2 h, 6 h. **(B)** GO analysis of common DEGs in AT-0 h/2 h, 6 h and ZM-0 h/2 h, 6 h. The sizes of the spots represent the number of genes, the colors represent the *p*-value and the shapes of the spots represent different GO pathways.

In this study, the genes with up-regulated expression in the salt-tolerant variety ZM-1 and down-regulated expression in the salt-sensitive variety AT were considered to be salt-tolerant genes. Common DEGs over the two time periods were selected in both varieties, and KEGG enrichment and GO analyses of the DEGs were performed. The results of the KEGG analysis showed that the major signaling pathways enriched in these genes included the MAPK signaling pathway, protein processing in the endoplasmic reticulum, endocytosis, and spliceosomes (**Figure 2A**). The GO analyses were divided into three categories: biological process (BP), molecular function (MF), and cellular component (CC). The results for MF showed that the main enriched pathways were heat shock protein binding, misfolded protein binding, and ATPase activity. The DEGs were mainly enriched in nucleus, cytoplasm, and nuclear cap-binding complex in CC. In terms of BP, the DEGs were mainly enriched in response to unfolded protein and cellular response to unfolded protein (**Figure 2B**).

### 3.3 Analysis of specific DEGs

**Figure 3:**
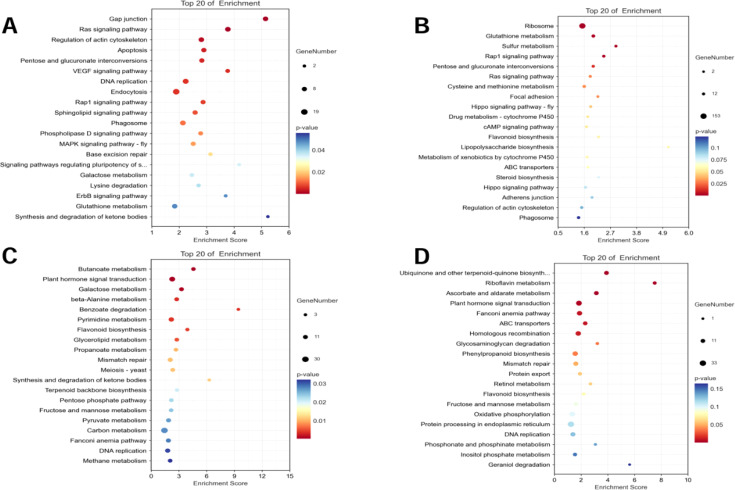
KEGG functional classification of specific DEGs. **(A)** AT-0/2 h, **(B)** AT-0/6 h, **(C)** ZM-0/2 h, **(D)** ZM-0/6 h. The sizes of the spots represent the number of genes, and the colors represent the *p*-value.

**Figure 4:**
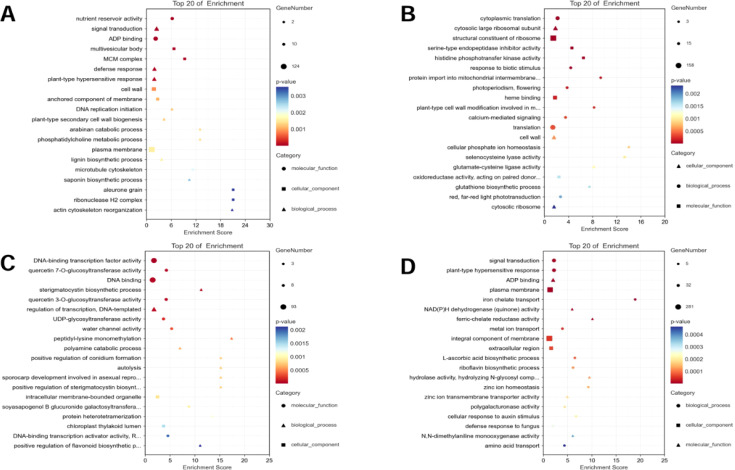
GO functional classification of the specific DEGs. **(A)** AT-0/2 h, **(B)** AT-0/6 h, **(C)** ZM-0/2 h, **(D)** ZM-0/6 h. The sizes of the spots represent the number of genes, the colors represent the *p*-value, and the shapes of the spots represent different GO pathways.

Specific DEGs to the two varieties at both time points were extracted and the KEGG and GO pathways were analyzed (**Figures 3**, **4**). For AT, the pathways that were significantly enriched in the KEGG analysis after 2 h of salt stress were gap junction, Ras signaling pathway, regulation of actin cytoskeleton, apoptosis, and pentose and glucuronate interconversions, among others (**Figure 3A**). The pathways that were significantly enriched after 6 h were ribosome, glutathione metabolism, sulfur metabolism, Rap1 signaling pathway, and pentose and glucuronate interconversions, among others (**Figure 3B**). The pathways that were significantly enriched in the salt-tolerant ZM-1 variety after 2 h of salt stress were butanoate metabolism, plant hormone signal transduction, galactose metabolism, beta-alanine metabolism, and benzoate degradation (**Figure 3C**), and the pathways that were significantly enriched after 6 h of salt stress were ubiquinone and other terpenoid-quinone biosynthesis, riboflavin metabolism, ascorbate and aldarate metabolism, plant hormone signal transduction, and Fanconi anemia pathway (**Figure 3D**).

GO analysis showed that the pathways that were significantly enriched in the salt-sensitive variety AT after 2 h of salt stress included nutrient reservoir activity, ADP binding, signal transduction, defense response, multivesicular body, and MCM complex (**Figure 4A**), and the pathways that were significantly enriched after 6 h of salt stress included response to biotic stimulus, cytosolic large ribosomal subunit, cell wall, structural constituent of ribosome, and serine-type endopeptidase inhibitor activity(**Figure 4B**). The pathways that were significantly enriched after 2 h of salt stress in the salt-tolerant variety ZM-1 were DNA-binding transcription factor activity, quercetin 7-O-glucosyltransferase activity, sterigmatocystin biosynthetic process, regulation of transcription, DNA-templated, intracellular membrane-bounded organelle, and chloroplast thylakoid lumen (**Figure 4C**), while the pathways that were significantly enriched after 6 h were signal transduction, plant-type hypersensitive response, ADP binding, NAD(P)H dehydrogenase (quinone) activity, plasma membrane, and integral component of membrane (**Figure 4D**).

### 3.4 Analysis of DEGs associated with salt tolerance

**Figure 5:**
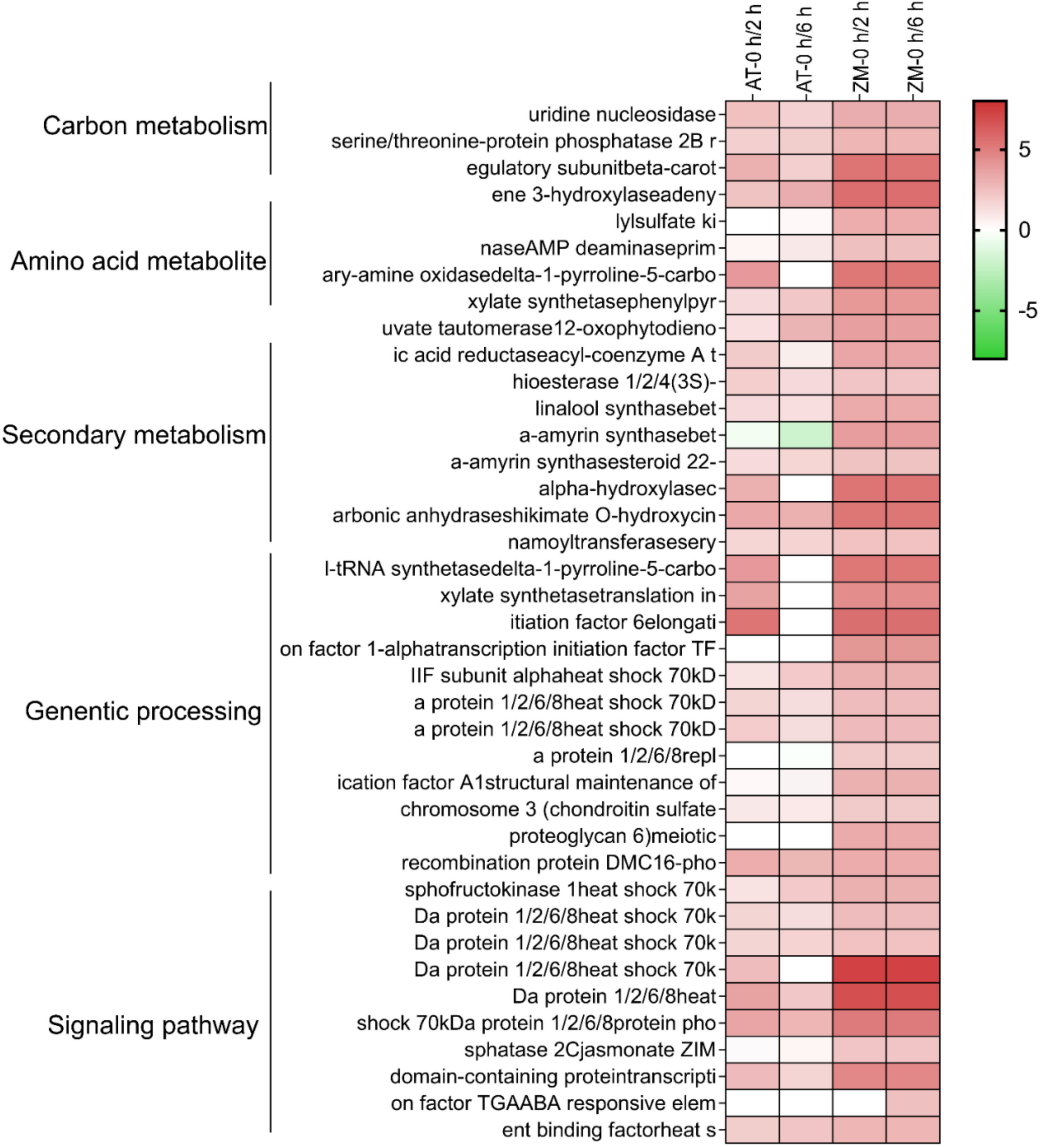
Heatmap of DEGs associated with salt tolerance: AT and ZM-1 seedlings at 2 h and 6 h of salt stress. Color scales indicate log_2_-transformed gene expression levels.

**Figure 6:**
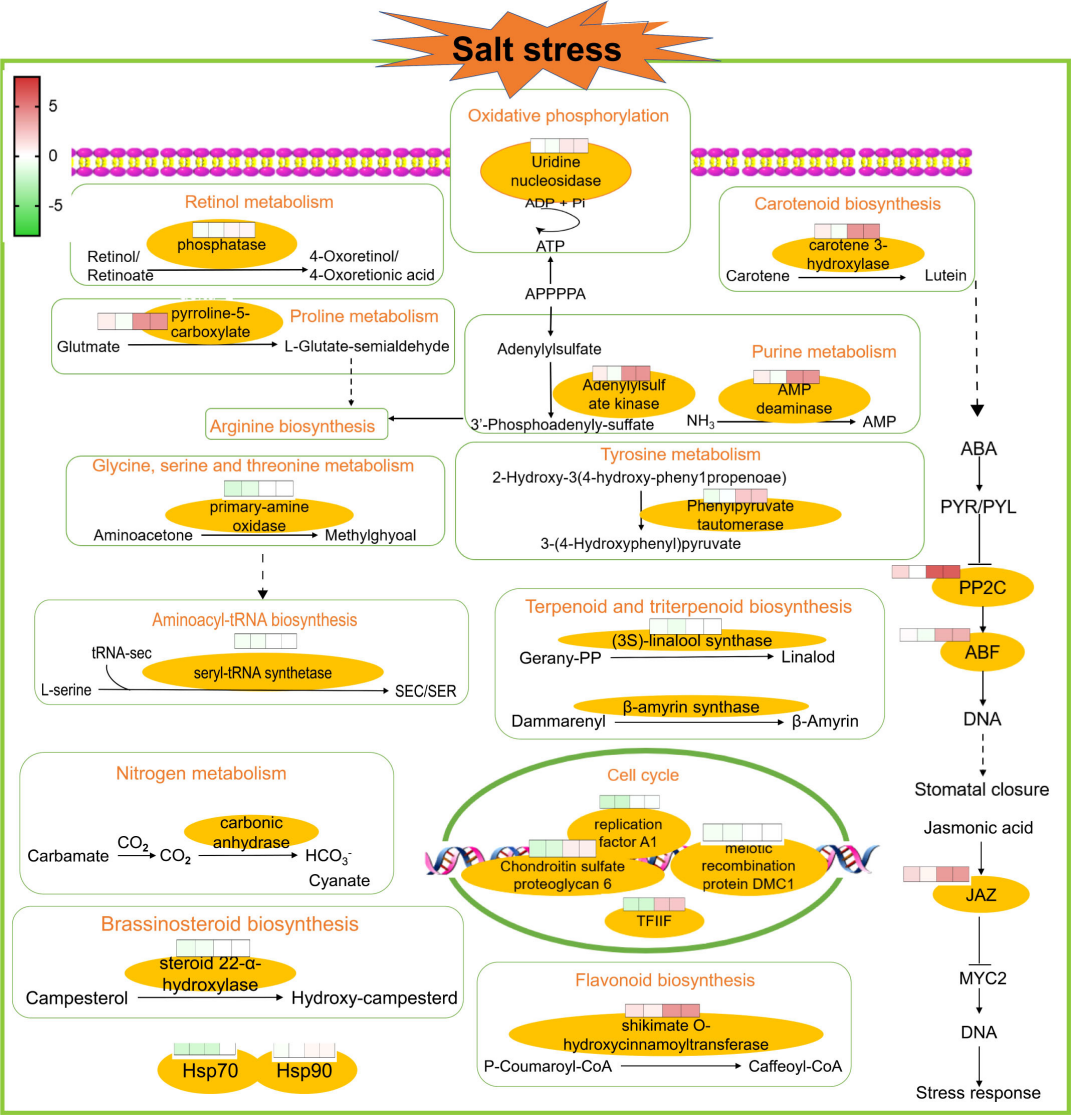
A map of the salt tolerance regulation gene network. Overview of salt regulatory network studies in AT and ZM-1 seedlings at 2 h and 6 h of salt stress.

In order to better explore alfalfa’s salt tolerance-related genes, it was assumed that under salt stress conditions, the genes related to salt tolerance would show higher expression in ZM-1, which has strong salt tolerance, than in the salt-sensitive variety AT. Therefore, the genes with up-regulated expression in ZM-1 and down-regulated or unchanged expression in AT were considered to be salt-tolerant genes. The log_2_(FC) values of the genes with similar expression patterns were averaged, and the genes were divided into five groups based on their gene functions: carbon metabolism, amino acid metabolites, genetic processes, secondary metabolism, and signaling pathway (**Figure 5**). These findings suggest that genetic processes and signaling pathways may play important roles in the salt tolerance response of alfalfa. In addition, to better explore the network regulatory relationships of the resistance genes, a map of the salt tolerance regulation gene network was drawn (**Figure 6**). The results showed that the AMPK signaling pathway, phytogenetic signaling pathway, terpene synthesis, and genes related to the cell cycle were significantly enriched in the salt-tolerant variety ZM-1 after 2 h and 6 h of salt stress, and the enrichment was higher than that of the salt-sensitive variety AT. The genes that were significantly enriched included beta-carotene 3-hydroxylase, adenylylsulfate kinase, carbonic anhydrase, shikimate O-hydroxycinnamoyl transferase, delta-1-pyrroline-5-carboxylate synthetase, elongation factor 1-alpha, heat shock 70kDa protein 1/2/6/8, protein phosphatase 2C, jasmonate ZIM domain-containing protein, and ABA-responsive element binding factor. The above genes may be candidates for improving salt tolerance in alfalfa.

### 3.5 Analysis of DEPs

**Figure 7:**
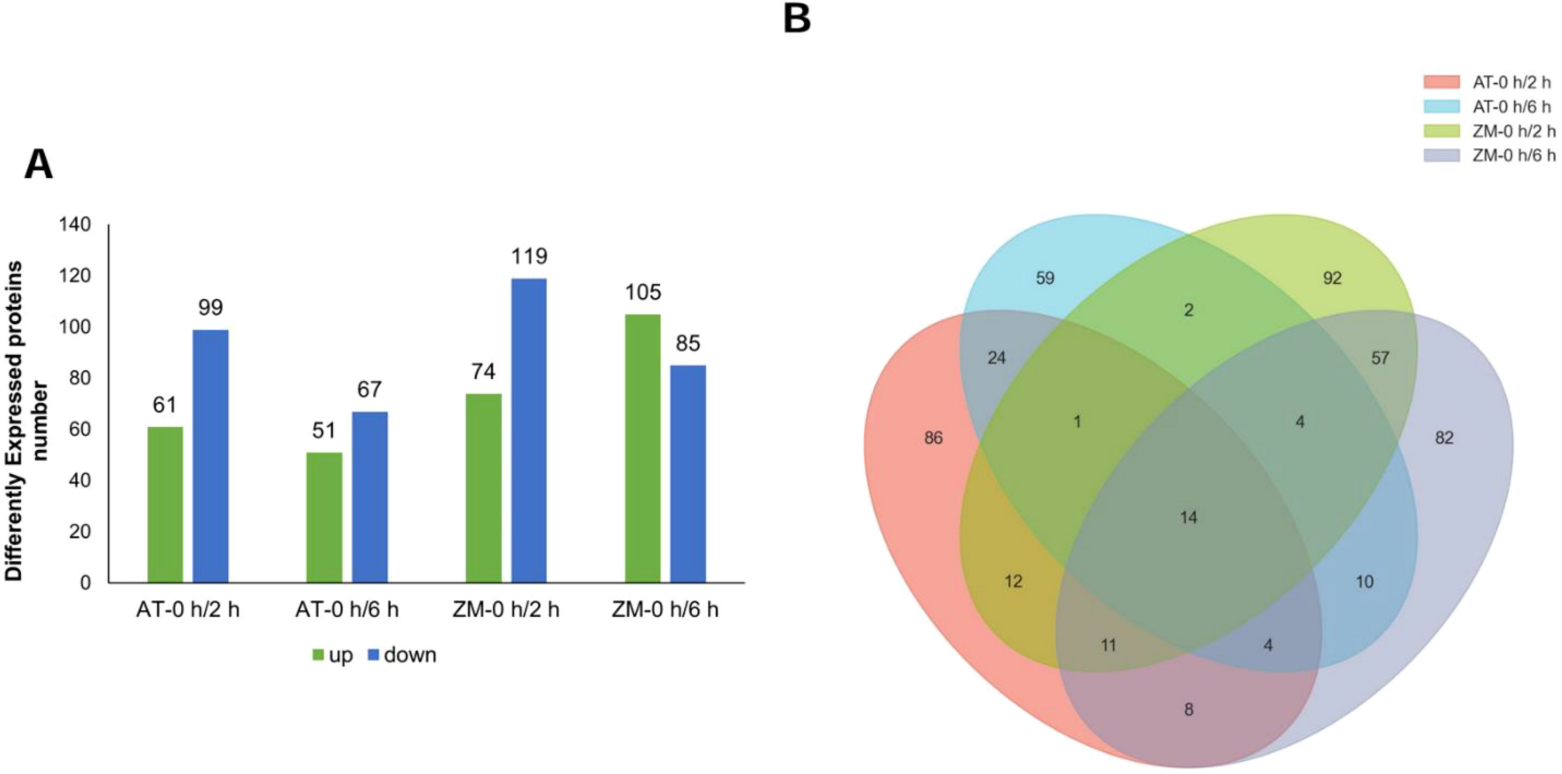
Analysis of DEPs. **(A)** The number of up-regulated and down-regulated proteins in the four groups. **(B)** The overlapped numbers of proteins in the four groups. AT-0 h/2 h and AT-0 h/6 h represent the comparison groups of the proteins identified at 2 h and 6 h after treatment to 0 h in Atlantic. ZM-0 h/2 h and ZM-0 h/6 h represent the comparison groups of the proteins identified at 2 h and 6 h after treatment to 0 h in Zhongmu-1.

Under salt stress for 2 h, 160 DEPs were screened in AT and 193 DEPs were screened in ZM-1, with more down-regulated proteins than up-regulated proteins in both varieties (**Figure 7A**). Among these, 38 DEPs were common to both varieties. Under salt stress for 6 h, 118 DEPs were detected in AT, among which, 51 DEPs were up-regulated and 67 DEPs were down-regulated. In contrast, 190 DEPs were identified in ZM-1, among which, 105 were up-regulated and 85 were down-regulated. The number of up-regulated proteins was 23.5% higher than the number of down-regulated proteins. Only 32 DEPs were common to both varieties after 6 h of salt stress. AT had 43 common DEPs after 2 h and 6 h of salt stress and 86 common DEPs were found in ZM-1 (**Figure 7B**).

### 3.6 GO annotations of the DEPs

**Figure 8:**
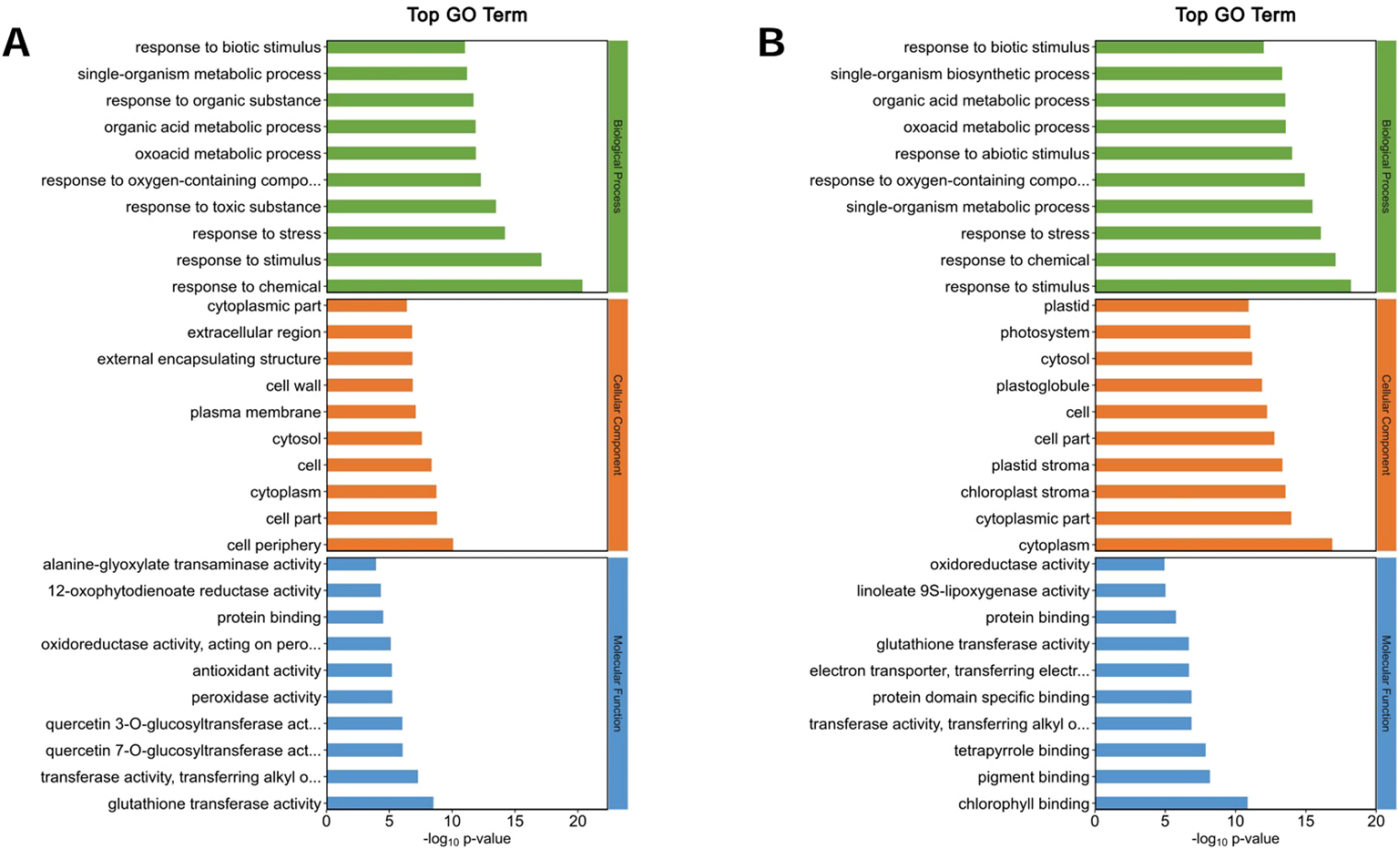
Go analysis of DEPs. **(A)** ZM-1 at 2 h compared with 0 h of salt stress. **(B)** ZM-1 at 6 h compared with 0 h of salt stress. GO pathways are categorized into three main groups: Biological Processes, Cellular Components, and Molecular Functions.

**Figure 9:**
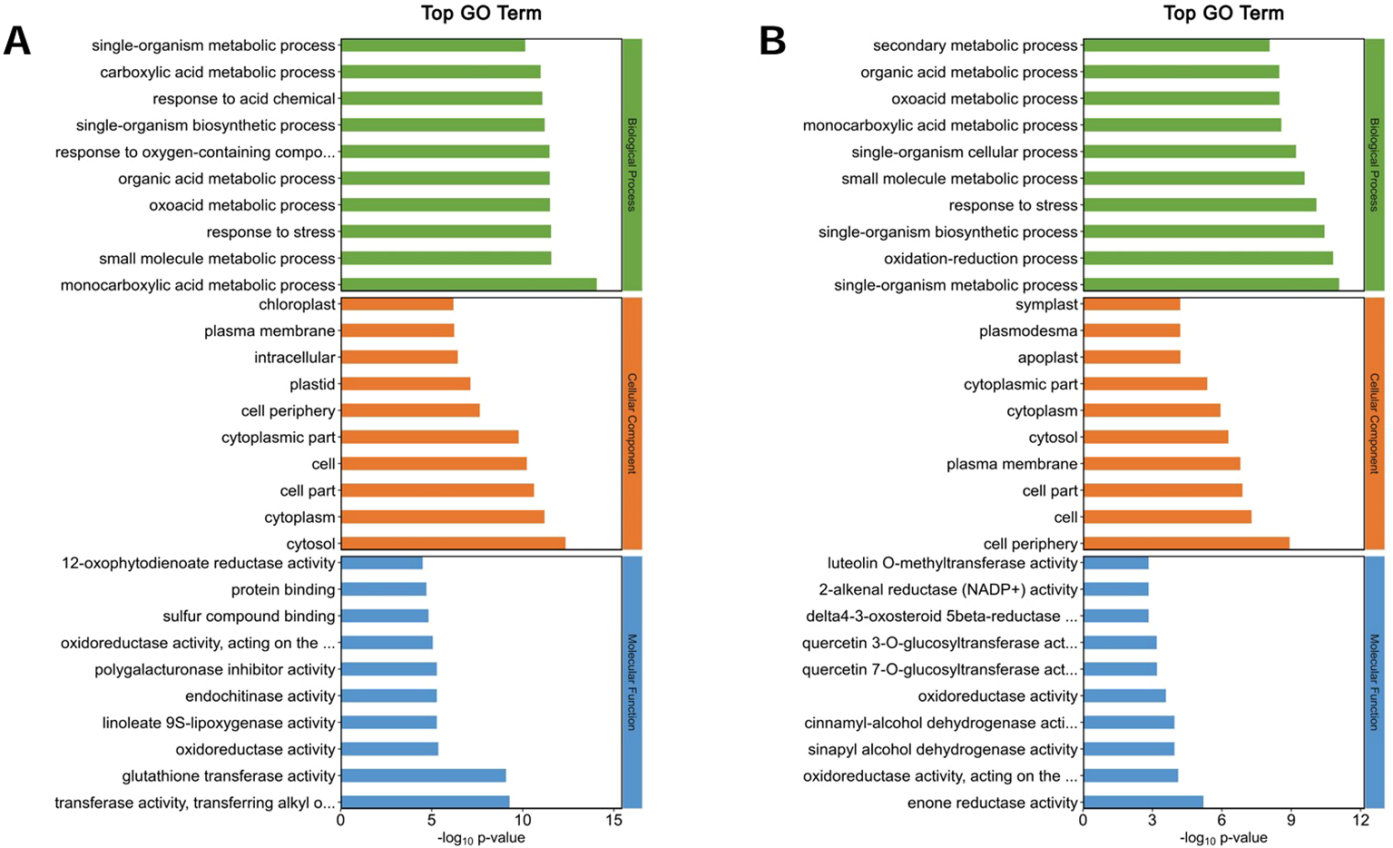
Go analysis of DEPs. **(A)** AT at 2 h compared with 0 h of salt stress. **(B)** AT at 6 h compared with 0h of salt stress. GO pathways are categorized into three main groups: Biological Processes, Cellular Components, and Molecular Functions.

The top 10 GO annotation classifications for the DEPs of ZM-1 and AT are shown in (**Figures 8**, **9**). In CC, the DEPs of the two varieties were significantly enriched in both cell and cell part under salt stress at the different time points. After 2 h of salt stress, extracellular region was enriched only in ZM-1, while intracellular region was only significantly enriched in AT. After 6 h of salt stress, plastid was enriched only in ZM-1, while cell periphery was only significantly enriched in AT. In the BP category, the most abundant subcategories enriched in both ZM-1 and AT were single-organism metabolic process and response to stress. Response to stimulus, response to chemical, etc. were only enriched in ZM-1. Small-molecule metabolic process and monocarboxylic acid metabolic process were only enriched in AT. In terms of MF, protein binding was present in both varieties after 2 h of salt stress, whereas after 6 h of salt stress, protein binding was present only in ZM-1.

### 3.7 KEGG pathway enrichment of the DEPs

**Figure 10:**
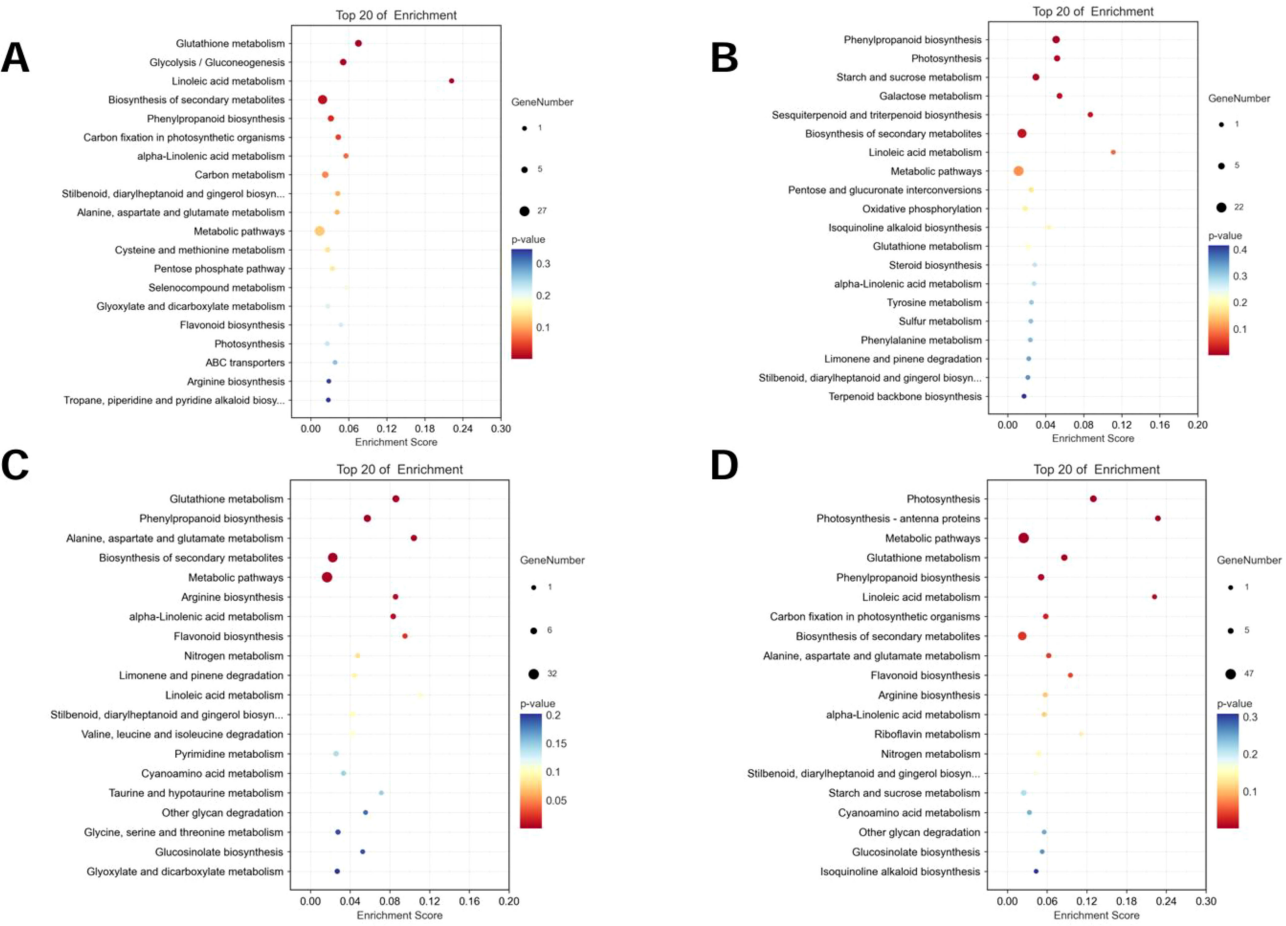
KEGG functional classification of DEPs. **(A)** AT-0/2 h, **(B)** AT-0/6 h, **(C)** ZM-0/2 h **(D)** ZM-0/6 h. The sizes of the spots represent the number of genes, and the colors represent the *p*-value.

To describe the complex biological behavior of the proteome, DEPs of ZM-1 and AT were subjected to KEGG pathway analysis. The DEPs in both varieties were mainly enriched in metabolic pathways. Notably, the enrichment of photosynthesis-related entries increased with prolonged salt stress in both species (**Figure 10**).

**Figure 11:**
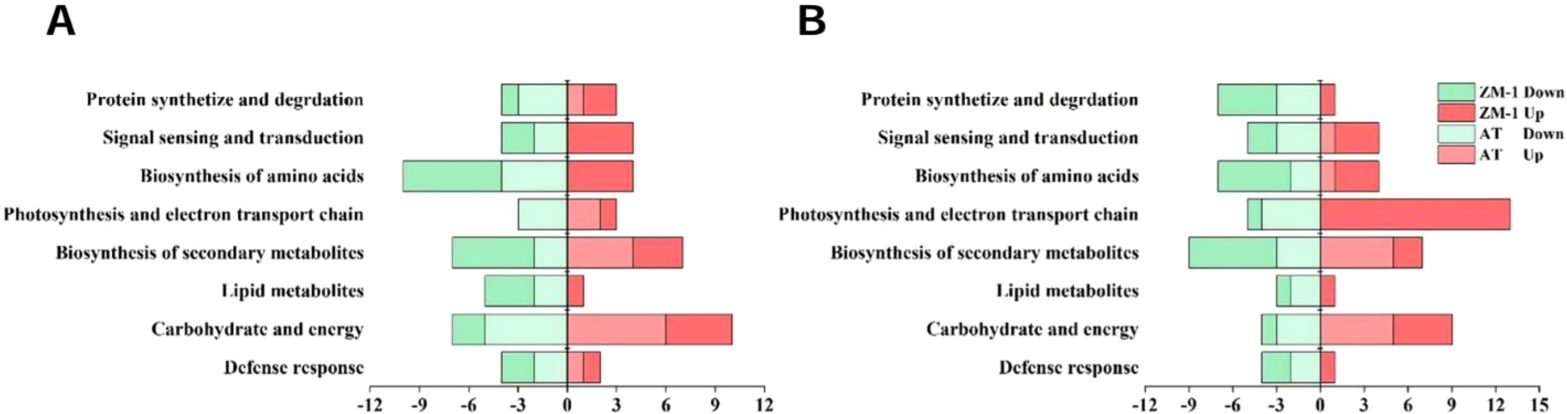
Functional classification of differentially regulated proteins. **(A)** AT and ZM-1 under salt stress for 2 h compared to 0 h. **(B)** AT and ZM-1 under salt stress for 6 h compared to 0 h.

In order to analyze the responses of AT and ZM-1 to different durations of salt stress at the protein level, the DEPs were classified into eight major categories based on the results of the KEGG analysis, namely, protein synthetize and degradation, signal sensing and transduction, biosynthesis of amino acid, photosynthesis and electron transport chain, biosynthesis of secondary metabolites, lipid metabolites, carbohydrates and energy, and defense response (**Figure 11**). For AT, no signaling sensing and transduction and amino acid biosynthesis proteins were up-regulated after 2 h of salt stress, but these began to be expressed over time. Moreover, the lipid metabolism-related proteins were down-regulated at both stress time points, and there were fewer down-regulated defense response-related proteins. The up-regulated proteins related to photosynthesis and electron transport chain proteins of ZM-1 were mainly enriched after 6 h of salt stress.

### 3.8 Transcriptome and proteome correlation analysis

**Figure 12:**
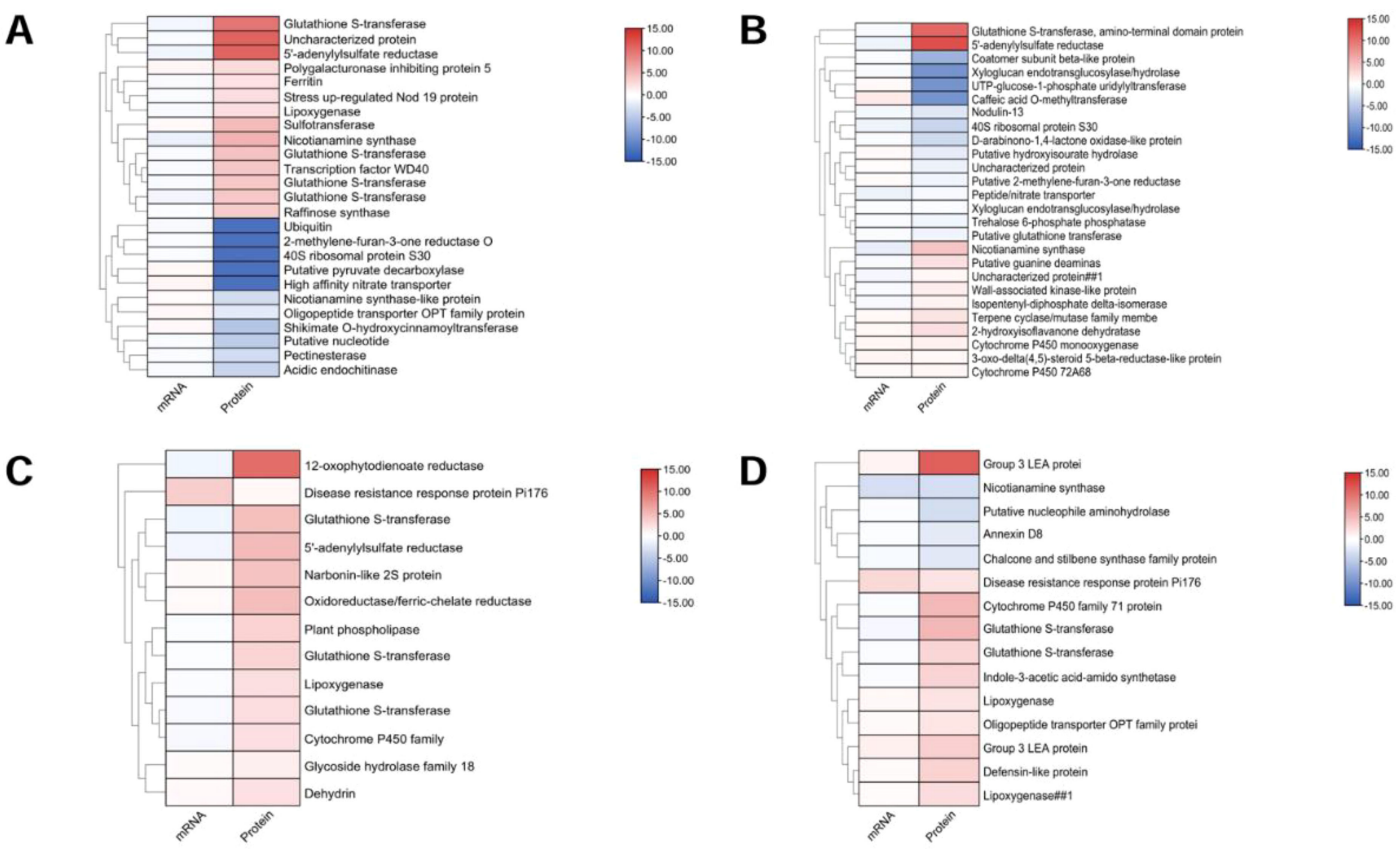
Functional classification of DEPs. **(A)**AT-0/2 h, **(B)** AT-0/6 h, **(C)** ZM-0/2 h and **(D)** ZM-0/6 h. Classification is based on biological processes obtained from GO classification and KEGG.

*Medicago truncatula* was chosen as the reference genome. Associate proteins with corresponding mRNAs based on annotation or id correspondence. The results showed that AT was associated with significantly more DEPs than ZM-1. Among them, genes associated with glutathione S-transferase were significantly expressed in AT after 2 h, and ZM-1 after both 2 h and 6 h, indicating that ZM-1 has a more durable antioxidant capacity after salt stress. The accumulation of 5’-adenylylsulfate reductase in AT was higher than that in ZM-1, suggesting that AT is more capable of promoting sulfur assimilation and increasing cysteine levels under salt stress. The 12-oxophytodienoate reductase-like protein exhibited significant accumulation in ZM-1 after 2 h of salt stress, which promoted jasmonate production and enhanced the plant’s stress tolerance. The LEA3-1 genes of the LEA family were significantly up-regulated in ZM-1, indicating that the osmotic adjustment function of ZM-1 was stronger under salt stress (**Figure 12**).

### 3.9 qRT-PCR validation

**Figure 13:**
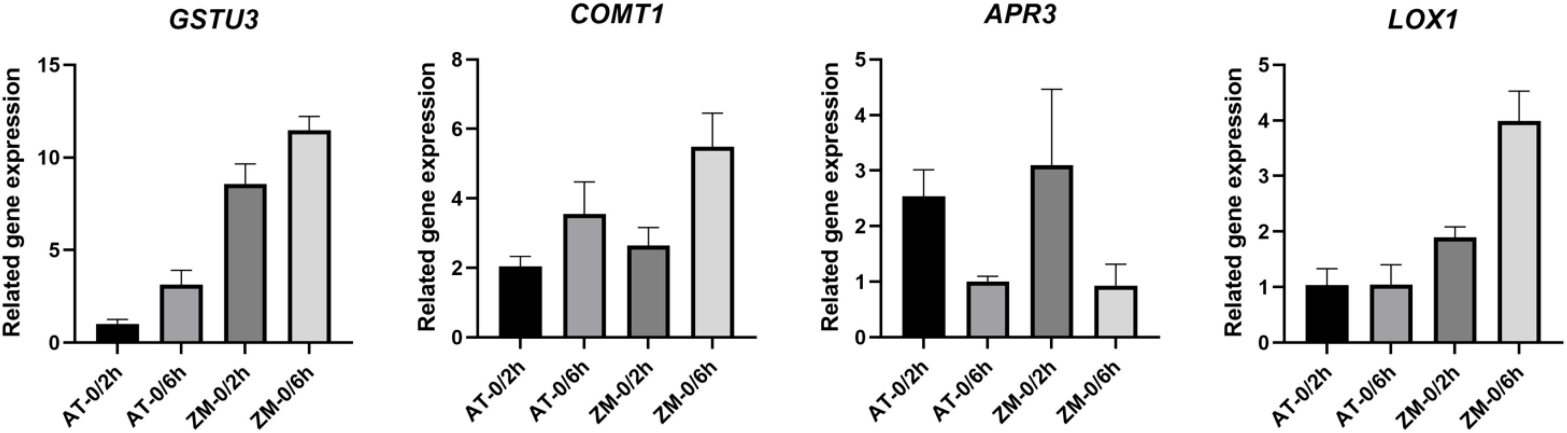
qRT-PCR validation of DEGs.

In order to verify the reliability of RNA-Seq data, four DEGs from melatonin biosynthesis genes, lipid metabolism, and defense response genes were selected for qRT-PCR validation. The results showed (**Figure 13**) that the qRT-PCR detection results were basically consistent with the trend of RNA-seq data, proving the accuracy and authenticity of RNA-Seq data.

## 4 Discussion

Salinity stress is one of the most significant abiotic stress factors that limit plant growth and yield enhancement. It causes osmotic stress, ionic toxicity, and oxidative stress, which ultimately inhibit plant growth and photosynthesis, obstruct nutrient uptake, and disrupt the normal growth and metabolism of plants (Arefian et al., 2019). Alfalfa is a cross-pollinated and homologous tetraploid, and its salt tolerance mechanism has not been intensively studied before. In recent years, transcriptomics, proteomics, and metabolomics analyses have been widely applied to the study of alfalfa plant growth and development and its response to abiotic stresses with the development of multi-omics technologies. This has led to new progress in the study of certain mechanisms in alfalfa (Ma et al., 2021, 2022; Wang et al., 2023). In this study, the DEGs and DEPs of two alfalfa varieties with differing salt tolerances under salt stress for 2 h and 6 h were compared. RNA-seq and TMT techniques were used to identify genes and proteins associated with salt stress. Overall, the number of salt-tolerant DEGs was significantly higher than that of DEPs, and the salt-tolerant variety ZM-1 exhibited a greater number of DEGs and DEPs with up-regulated expression after 6 h of salt stress. This suggests that these DEGs and DEPs are likely related to salt tolerance. The screened DEGs and DEPs were categorized into different functional groups.

### 4.1 Signal sensing and transduction

Plants regulate gene expression through complex abiotic signaling pathways, leading to the expression of reactive proteins in adverse environments (Zhang et al., 2023). Plant hormones play an important role in regulating plant signaling. In general, abscisic acid (ABA) inhibits leaf senescence. ABA signaling induces stomatal closure, which is now a common expression of stress tolerance, and the ABA response element binding factor genes *ABR17*, *GEM-like protein 5*, *PYL12*, etc. are significantly up-regulated after salt stress, with the salt-tolerant cultivar ZM-1 accumulating significantly more than the salt-sensitive cultivar AT. Studies have shown that ABA treatment can significantly alleviate the oxidative damage induced by salt stress on tomato seedlings, increase the protective enzyme activity of cells, and effectively reduce the accumulation of Na^+^ in the aboveground part of the plant, thus maintaining a higher K^+^/Na^+^ ratio in the plant (Khadri et al., 2007). In addition, ABA induces enhanced activity of plasma membrane ATPase and vesicular membrane ATPase in cucumber roots under salt stress; these ATPases act as proton pumps to provide a greater driving force for Na^+^/H^+^ reverse transporter proteins to promote exocytosis and ion compartmentalization of Na^+^. This mitigates the toxicity of high Na^+^ to the plant (Janicka-Russak et al., 2012). Therefore, the high expression of these types of genes in alfalfa can improve the salt tolerance of the plant.

Moreover, the transcriptomic results revealed that the heat shock protein 70 family (*HSP70*), which is involved in the MAPK signaling pathway, was also significantly enriched in the salt-tolerant cultivar ZM-1. The MAPK signaling pathway plays a key role in a variety of processes, including plant growth, development, and response to abiotic stresses (Colcombet and Hirt, 2008). It has been shown that almost all stress responses induce the production of heat shock proteins (HSPs), whose transcription is regulated by heat shock factors (HSFs). HSPs act as molecular chaperones to assist in the correct assembly or folding of proteins, preventing irreversible aggregation of proteins and thus maintaining cellular homeostasis under various adverse conditions. In terms of regulation, HSPs are involved in a variety of biological functions such as the degradation of target proteins and protein transmembrane transport processes (Luo et al., 2020). İlker Büyük studied the HSP70 family in common bean did a genome-wide identification to demonstrate the response of the gene to salinity (Büyük et al., 2016). The main function of *HSP70* is to participate in the folding of nascent proteins, but it can also bind to some protein hydrophobic regions to prevent protein polymerization and can participate in the transmembrane transport of proteins and the degradation process of target proteins. *HSP70* can be induced by all stresses leading to the denaturation of proteins and plays an important role in the case of exposure to a wide range of abiotic stresses (Usman et al., 2017). In this study, the *HSP70*, *HSP70-1*, etc. genes were expressed in ZM-1 after salt stress, implying that the protein complexity and stability of this variety may be improved.

Ribosomal proteins are essential components for protein synthesis and play an important role in defense against harsh environments. The expression of *RPS30A*, located in the small subunit, was reduced in both species, inhibiting protein synthesis. Further, NBS-LRR proteins are important intracellular receptors that directly or indirectly recognize pathogen-effector proteins and are involved in signal transduction under abiotic stress (Li et al., 2017). Compared with the salt-sensitive variety AT, NBS-LRR protein genes such as *RPM1* and *RGA1* were expressed at an earlier stage in ZM-1, suggesting that salt-tolerant varieties can sense salt stress earlier.

Fructose-1,6-bisphosphate is an important metabolic enzyme involved in glycolysis/gluconeogenesis and the Calvin cycle (Patipong et al., 2019), whereas 6-phosphofructokinase is a novel regulator indispensable for early chloroplast development (Zhu X. et al., 2020). The overexpression of 6-phosphofructokinase alters growth, photosynthesis, and stress responses in higher plants (Lu et al., 2012). The 6-phosphofructokinase gene was involved in the AMPK signaling pathway in this study. AMPK signaling, as an important kinase-regulating energy homeostasis, is one of the central regulators of cellular and organismal metabolism in eukaryotic organisms and is responsible for maintaining the smooth functioning of cellular physiological activities. Therefore, the 6-phosphofructokinase gene may also be related to salt tolerance in alfalfa.

### 4.2 Carbohydrate and energy metabolism

Carbohydrate metabolism is a major transition library in the sugar accumulation pathway (Zhu et al., 2017). The *UGP1*, *TPPA*, *PME7*, *CWINV1*, *BGLU11*, and *SBE2.2*, genes, related to starch and sucrose metabolism, were expressed in alfalfa after 6 h of salt treatment. The expression of these proteins promotes the accumulation of soluble sugars, which provide substrates and energy for orderly metabolism in the salt environment (Zhu Y. et al., 2020). Oxidative phosphorylation is an important pathway for bacterial generation of adenosine triphosphate (ATP). ATP, which is made up of adenine, ribose, and three phosphoryl groups, releases a large amount of energy upon hydrolysis and is the most direct source of energy in living organisms. Studies have shown that carotenoid pigment is essential for plants to adapt to saline and alkaline environments (Liu et al., 2020). Thus, the oxidative phosphorylation genes *ATPI* and *AHA8* and the carotenoid biosynthesis gene *CCD1* may be candidate genes for improving salt tolerance in alfalfa. However, their ability to improve salt tolerance in alfalfa needs to be further verified.

### 4.3 Defense response

Pathogenesis-related class 10 (PR-10) proteins are highly conserved plant proteins that are induced in response to abiotic and biotic stress factors (Agarwal and Agarwal, 2014). PR-10 is primarily a cell membrane protein expressed in several plant tissues such as the roots, stems, flowers, and fruits in some plant species. The expression of this protein is up-regulated under abiotic and biotic stress conditions such as bacteria and fungi, cold, salinity, drought, oxidative stress, and physical injury. In the current study, the disease resistance response protein Pi176 was significantly enriched after salt treatment in alfalfa, suggesting that this protein plays a general role in the plant’s defense mechanisms.

Many studies have found that partially reduced or activated oxygen derivatives are highly active and toxic compared to O_2_ and can disrupt cellular oxidation, which is a consequence of the development of efficient ROS scavenging mechanism by aerobic organisms (Inupakutika et al., 2016; Jin et al., 2009). The ROS scavenging mechanism in plants under salt stress is an important part of the plant’s salt tolerance mechanism. Most of the genes involved in antioxidant stress during different periods of salt stress treatment in this study were peroxidase-like genes, including POD and glutathione peroxidase (GSH-Px). The most common antioxidant enzymes POD and GST were differentially expressed after 2 h and 6 h of salt stress in ZM-1. Numerous studies have shown that the expression of POD and GST can alleviate the damage caused to the plant under stress and increase the antioxidant capacity (Ji et al., 2019). By comparing different varieties of alfalfa (Gongnong No.1 and Sibeide), Gao et al. found that under salt stress, GST, POD related genes were significantly up-regulated in salt-tolerant varieties (Gongnong No.1) and down-regulated in salt-sensitive varieties (Sibeide) (Gao et al., 2024). In this study, the expression of GST proteins was significantly up-regulated in both varieties after different durations of salt stress, and the genes expression levels were correlated. The *GSTL3*, *GSTU3*, *GSTU7*, *GSTU8*, *GSTU19*, etc. genes were differentially expressed after different durations of salt stress treatment. This may contribute to the alleviation of the damage caused by high salt stress by removing the higher level of ROS in the plant cells under salt stress. In addition, the proteomics results showed that *PER5*, *PER10*, *PER15*, and *PER51* were up-regulated in ZM-1 after 2 h and 6 h of salt stress, respectively, while *PER10*, *PER47*, and *PER21* were down-regulated in AT. These results indicate that PODs removed the oxidized products of the salt-tolerant variety under salt stress and mitigated the salt stress damage.

Salt stress leads to an imbalance in ionic homeostasis, which can ultimately lead to inhibition of plant growth, decreased yields, and accumulation of excess Na^+^ in the cytosol, so maintaining intracellular ionic homeostasis is an important aspect of salt acclimation (Liu et al., 2014). It has been shown that different gene families of high-affinity nitrate transporter (NTR) are differentially expressed under stress conditions, but this is sufficient to justify their possible involvement in the stress response (Pu et al., 2023). Similarity, in tomato (*Solanum lycopersicum* L.), the *SlNRT2* family of proteins is also involved in the response to drought and salinity (Akbudak et al., 2022). We found that *NTR2.4* was significantly up-regulated after salt stress in ZM-1, indicating that this gene responds to salt stress and may be used as a candidate gene for salt tolerance for further study.

The cytochrome P450 (CYP) superfamily is the largest family of enzyme proteins in plants. Members of this superfamily are involved in a variety of metabolic pathways with unique and complex functions. The CYP family is essential for the production of secondary metabolites, antioxidants, and phytohormones in higher plants, which thus act as growth and developmental signals or protect plants from various biotic and abiotic stresses. CYP proteins have been found to bind to the membranes of organelles such as the endoplasmic reticulum, Golgi apparatus, and mitochondria, and are a class of hydrophobic and heme-containing binding proteins (Chen et al., 2021). CYP family proteins catalyze the oxidation of various substrates through the activation of oxygen molecules (Mao et al., 2013) and, according to Maria Carelli, the CYP monooxygenase *CYP716A12* is involved in the biosynthesis of saponins in the defense mechanisms of plants (Carelli et al., 2011). In this study, *CYP716A1* and *CYP72A68* in ZM-1 were significantly up-regulated after 6 h of salt treatment. Moreover, the results of the transcriptome and proteome analyses showed that the gene and protein were significantly up-regulated after 6 h of salt stress in AT. This suggests that the plant may resist stress by activating the biosynthetic pathway of triterpenoid saponins.

### 4.4 Lipid metabolites

Fatty acids are the main components of biological membranes. Unsaturated fatty acids play an important role in determining the physiological properties of biological membranes. Increasing the degree of unsaturation of fatty acids can increase the fluidity of membrane lipids. Many studies have found that the content of unsaturated fatty acids in membrane lipids is closely related to many stresses (Nian and Chen, 2012). The transcriptome data in this study showed that the expression levels of genes related to unsaturated fatty acid biosynthesis, especially α-linolenic acid metabolism and *OPR1, OPR2*, and *DOX1*, were higher in ZM-1 than in AT. This suggests that the high expression of unsaturated fatty acid genes can improve the stress tolerance of alfalfa.

Lipoxygenase (LOX), a member of the non-heme iron-dioxygenase family, catalyzes the synthesis of oxidized lipids and their derivatives and plays an important role in the stress response. LOX is commonly found in plant organs such as the roots, stems, leaves, flowers, fruits, and seeds, and is expressed differently in plants at different stages of development. The diversity of positioning implies that there will also be some variability in the function of LOX. For example, *DkLOX3* in persimmon is mainly localized in the cytoplasm, mitochondria, and plastids and regulates fruit development (Hou et al., 2015). Wang et al. found that genes (LOX, OPR) involved in jasmonic acid synthesis were significantly up-regulated after salt stress in alfalfa, suggesting that salt stress causes an increase in JA to enhance plant resilience (Wang et al., 2023) In this study, the expression levels of *LOX1* and *LOX5* were up-regulated after 6 h of salt stress in ZM-1, suggesting that the accumulation of this family of genes enhances plant stress tolerance.

### 4.5 Photosynthesis and the electron transport chain

The effects of salt on photosynthesis are mainly characterized by the inhibition of CO_2_ diffusion to chloroplasts and changes in leaf photochemistry (Lovelli and Perniola, 2014). Chloroplasts are more sensitive to salt stress than other organelles (Yousuf et al., 2016). Salt stress affects the abundance of proteins related to electron transport, photosystem I (PSI), photosystem II (PSII), ATP synthase, and carbon fixation. Chlorophyll a-b binding protein (CAB) is involved in light harvesting and solar energy transfer and is one of the most abundant proteins in chloroplast-like vesicles (Kong et al., 2016). In this study, the *CARCAB1* gene was significantly up-regulated in ZM after 6 h of salt stress. The light-harvesting complex (LHC) is composed of photosynthetic proteins and pigments and is an important functional element of chloroplasts. In higher plants, one of the main components of LHC is CAB (Li et al., 2020). PSI exists *in vivo* as a trimer and a monomer and has been found to have the most complex membrane protein structure. A single unit of PSI consists of 127 cofactors covalently bound to a variety of different proteins (PsaA, PsaB, PsaC, PsaD, PsaE, PsaF, PsaG, and 16 other proteins). The cofactors are not only crucial for the proteins to function but also play an important role in the assembly and structural integrity of PSI. The current study revealed that the expression levels of the *psbA*, *psbB*, *psbD*, *psaB*, *psaC*, *PSBP1*, *LHCB2.1*, *LHCB3*, and *LHCB5.1* genes, among others, were up-regulated in ZM-1 under salt stress for 6 h. This indicates that these genes may serve as candidate genes for plant salt tolerance. Many studies have shown that high LHC expression mitigates the damage caused by salt stress.

Ferredoxin (Fd), a soluble chloroplast protein in the ferredoxin/thioredoxin system, is capable of sensing changes in the redox potential and converting electronic signals into biochemical signals. In the presence of light, Fd is reduced and transfers electrons to ferredoxin-NADP^+^ oxidoreductase (FNR) (Dai et al., 2004). *LFNR1* is up-regulated in salt-tolerant species.

CO_2_ assimilation is a carbon sequestration reaction during photosynthesis that converts the reactive chemical energy in ATP and NADPH into stable chemical energy. After salt treatment, the expression levels of *rbcL*, *CYFBP*, and *RBCS-3B* in alfalfa were up-regulated.

Overall, the above results indicate that the photosynthetic capacity of AT is sensitive to salinity. Similar to the results of the current study, a comparative proteomic analysis of seedlings of two rice varieties (salt-tolerant Pokkail and salt-sensitive IR64) also showed that Pokkail had better photosynthetic mechanisms to cope with salt stress (Lakra et al., 2019).

### 4.6 Biosynthesis of secondary metabolites

The caffeic acid O-methyltransferase (*COMT*) *COMT1* was highly expressed in AT and ZM-1 after 6 h of salt stress. *COMT* has been shown to catalyze the conversion of N-acetylserotonin to melatonin. This process is thought to regulate melatonin synthesis. Wang et al. note that the role of the mango *COMT* gene (*MiCOMT1*) in regulating salt stress tolerance, and confirmed that *COMT1* is a positive regulator of salt tolerance to abiotic stress in plants (Wang et al., 2024). In addition, Li et al. found that *COMT* expression was up-regulated after salt treatment of alfalfa, a result that suggests that melatonin is closely related to salt tolerance in alfalfa (Li et al., 2022).

The 5’-adenylylsulfate reductase *APR3*, which is associated with the sulfur/sulfate metabolic pathway, was down-regulated in the two alfalfa varieties in this study, suggesting reduced synthesis of thioglucoside and methionine in the root samples.

The terpenoids family of plant secondary metabolites is widely distributed in archaea, bacteria, and eukaryotic organisms. The family is the most diverse and structurally varied class of natural products in plants and mainly includes hemiterpenes, monoterpenes, sesquiterpenes, diterpenes, sesterterpenes, triterpenes, etc. These are produced through the mevalonate pathway (MVA) and methylerythritol 4-phosphate pathway (MEP) and are generated with specific structures and functions. For example, steroids act as hormone signals, carotenoids as antioxidants in plant photosynthesis, and quinones as electron transporters (Fan, 2020). In this study, a large number of monoterpene, sesquiterpene, and triterpene synthesis-related genes, including *SQE1*, *BAS*, and *HMG1*, were found to be up-regulated in alfalfa, suggesting that this class of compounds could serve as candidate genes related to salt tolerance in alfalfa.

### 4.7 Genes associated with DNA replication and repair

Cells respond to DNA damage by activating a robust DNA damage response pathway. This provides sufficient time for specific DNA repair pathways to physically remove the damage in a substrate-dependent manner. In this study, a large number of genes related to DNA replication and repair, such as *DRT100*, *MCM7*, *PSF2*, *SLD5*, *dpd11*, and *PSF1*, were found to be up-regulated in the salt-tolerant cultivar ZM-1. To date, five DNA damage pathways have been reported, including base combination repair, nucleotide recombination repair, mismatch repair, homologous recombination repair, and non-homologous end joining (Chatterjee and Walker, 2017). Salt stress has been found to have important effects on cells involved in DNA replication (Geng et al., 2020). Wei et al. examined the salinity tolerance of alfalfa and found one CAR gene (*MS.gene031323.t1*) that was simultaneously involved in DNA replication, mismatch repair, nucleotide excision repair, and homologous recombination (Wei, 2022). This suggests that the genes significantly up-regulated in the salt-tolerant cultivar ZM-1 in the present study may serve as candidate genes for improving alfalfa’s salt tolerance.

## 5 Conclusions

In this study, a greater number of DEGs were detected after 6 h of salt stress as compared to after 2 h of salt stress in both the salt-tolerant and salt-sensitive alfalfa varieties. However, the numbers of DEPs screened in AT and ZM-1 were both reduced after 6 h of salt stress as compared to 2 h of salt stress. Moreover, the reduction in DEPs screened in AT was greater than that screened in ZM-1. A comparison of the transcriptomes and proteomics of the two varieties revealed that photosynthesis-related proteins, including *LHC*, *CAB*, *LFNR1*, and *rbcL*; genes related to the biosynthesis and metabolism of secondary metabolites, including 5’-adenylylsulfate reductase *APR3*, terpene biosynthesis-related genes such as *SQE1*, *BAS*, and *HMG1*, and the caffeic acid O-methyltransferase *COMT1*; proteins and genes involved in plant signal transduction, including the *HSP70* family and the ABA-binding response factor *ABR17*; the linolenic acid synthesis genes *OPR1* and *DOX1* and the LOX family, which are involved in lipid metabolism; PR-10, the GST family, the PER family, and the CYP family, which are involved in the defense response; the carotenoid biosynthesis-related gene *CCD1*; the oxidative phosphorylation-related genes *ATPI*, and *AHA8*; and a large number of genes involved in DNA replication and repair, including *DRT100*, *MCM7*, *PSF2*, and *SLD5*, are up-regulated in alfalfa after exposure to salt stress. It is expected that further research on the above differential genes and proteins will lay the foundation for salt tolerance breeding in alfalfa.

## Data Availability

The data presented in the study are deposited in the NCBI repository, accession number PRJNA1182104.
