# Supplementary material for: Comparative transcriptomic and proteomic analyses of two salt-tolerant alfalfa (Medicago sativa L.) genotypes: investigation of the mechanisms underlying tolerance to salt
Source: Front Plant Sci. 2024 Nov 13;15:1442963. doi: 10.3389/fpls.2024.1442963 (PMC11598528; doi:10.3389/fpls.2024.1442963)
Supplement: Supplementary file 1 [file Table1.docx]

Supplementary Material

# Supplementary Tables

Table.S1. Primer sequence for qRT-PCR validation

| Gene ID | Forward primer | Reverse primer |
| --- | --- | --- |
| *GSTU3* | TCTGGCCATGTCCACAAACA | TGGAATCAAACCAAAGGGGTTC |
| *COMT1* | GCCCCTTCCTATCCTGGAGT | GATGGCATCTGCTTTAGGCAC |
| *APR3* | CGTTTGTCCCAAGGAATCGC | GAAGCCTCATCTCCCTGGGT |
| *LOX1* | TTTCCGCACTGATGGTGAACA | TGCAGATTTGCTGACTCTGAT |
| *β-actin* | CAAAAGATGGCAGATGCTGAGGAT | CATGACACCAGTATGACGAGGTCG |
